# Current marijuana use is associated with lower circulating α-Klotho levels in US adults even after adjusting for tobacco use: A national cross-sectional analysis of NHANES

**DOI:** 10.18332/tid/208001

**Published:** 2025-07-25

**Authors:** Kai Wei, Xiaotong Chen

**Affiliations:** 1 Department of Pharmacy, Guizhou Provincial People's Hospital, Guiyang, China; 2 Department of Laboratory Medicine, Jing’an District Central Hospital of Shanghai, Jing’an Branch Affiliated to Huashan Hospital, Fudan University, Shanghai, China

**Keywords:** marijuana, tobacco, α-Klotho, biological aging

## Abstract

**INTRODUCTION:**

α-Klotho is a circulating protein linked to longevity and healthy aging. While tobacco use is known to reduce α-Klotho levels, the effects of marijuana use on this aging-related biomarker remain unclear. This study aimed to examine the association between marijuana use and serum α-Klotho levels in a nationally representative sample of US adults.

**METHODS:**

We conducted a secondary analysis of publicly available data from the National Health and Nutrition Examination Survey (NHANES) 2007–2016, including 6601 adults aged 40–59 years with available serum α-Klotho levels and complete substance use data. Multivariable linear regression models were used to estimate the association between marijuana use and α-Klotho levels.

**RESULTS:**

Current marijuana use was significantly associated with lower serum α-Klotho levels compared to never use (β= -0.084; 95% CI: -0.126 – -0.041), independent of tobacco and illicit drug use. In the combined exposure model, those using both marijuana and tobacco exhibited the greatest reduction in α-Klotho (β= -0.112; 95% CI: -0.162 – -0.062). The inverse association persisted across most subgroups, including both current smokers (β= -0.078; 95% CI: -0.134 – -0.021) and non-smokers (β= -0.087; 95% CI: -0.151 – -0.002).

**CONCLUSIONS:**

Marijuana use was independently associated with lower circulating α-Klotho levels, suggesting a potential link between cannabis exposure and accelerated biological aging, even after adjusting for tobacco use. These findings highlight the need for further longitudinal studies to investigate the long-term impact of marijuana and tobacco use on systemic aging processes and health effects.

## INTRODUCTION

The anti-aging protein α-Klotho is associated with longevity and with age-related traits such as cardiovascular disease, cognitive decline, and pulmonary conditions, among others^1,2^. Its circulating levels are influenced by various environmental and lifestyle factors, with tobacco exposure being particularly notable^3^. In contrast, the impact of marijuana use on systemic aging remains largely unexplored, despite epidemiological data indicating a rising prevalence among US adults^4^. Given cannabis’s potential to induce oxidative stress and inflammation^5^, it may influence aging-related biomarkers such as α-Klotho.

We aimed to investigate the association between marijuana use and circulating α-Klotho levels in a nationally representative sample of US adults using data from the National Health and Nutrition Examination Survey (NHANES), while taking into account tobacco use.

## METHODS

### Data source and participants

We conducted a secondary analysis using publicly available and pooled data from five consecutive cycles of NHANES spanning 2007 to 2016. NHANES employs a stratified, multistage probability sampling design to produce a nationally representative sample of the non-institutionalized US civilian population. Within this dataset, 17345 participants aged 12–59 years completed the illicit drug use questionnaire, and 13766 individuals aged 40–79 years provided blood samples for serum α-Klotho measurement. Our analysis focused on a subsample of 6601 participants who had both available α-Klotho data and complete tobacco and substance use information. NHANES protocols were approved by the National Center for Health Statistics Research Ethics Review Board, and all participants provided written informed consent. This study was conducted in accordance with the Strengthening the Reporting of Observational Studies in Epidemiology (STROBE) guidelines^6^ (Supplementary file Table 3).

### Measures

Current marijuana use was defined as participants who responded ‘yes’ to the question ‘Ever used marijuana or hashish?’ (DUQ200) and reported using marijuana within the past 30 days, as determined from variables DUQ220Q and DUQ220U (‘Last time used marijuana or hashish’). Ever marijuana use was defined as a ‘yes’ response to DUQ200 and last use occurring more than 30 days prior to the survey. Never marijuana use was defined as a ‘no’ response to DUQ200. Tobacco use was defined as answering ‘every day’ or ‘some days’ to the question ‘Do you now smoke cigarettes?’ (SMQ040), or having serum cotinine levels >10 ng/mL. Illicit drug use was defined as participants who answered ‘yes’ to the question ‘Ever used cocaine, heroin, or methamphetamine?’ (DUQ240) and reported use of these substances within the past 30 days. Serum α-Klotho levels were measured using a validated commercial ELISA kit and log-transformed to approximate normal distribution. Covariates included demographic factors, socioeconomic status, lifestyle factors, and medical conditions potentially influencing both α-Klotho^2^ and cannabis use behaviors^7^.

### Statistical analysis

Multiple imputation was used to handle missing covariate data. Survey-weighted multivariable linear regression was used to assess the association between marijuana use and circulating α-Klotho. Subgroup analyses were conducted to explore potential heterogeneity of associations. All analyses were performed using R version 4.3.1 (R Foundation for Statistical Computing), with a two-sided p<0.05 considered statistically significant.

## RESULTS

A total of 6601 participants aged 40–59 years were included in the analysis, representing an estimated 63.6 million US community-dwelling adults. The median age was 49 years (interquartile range: 45–54); 51.6% were female, with 71.6% non-Hispanic White participants. Overall, 9.5% of participants reported marijuana use within the past 30 days, and 50.0% had ever smoked marijuana. Demographic and clinical characteristics stratified by marijuana use status are comprehensively detailed in Supplementary file Table 1.

In multivariable adjusted models, both ever and current marijuana use were significantly associated with lower serum α-Klotho levels (Table 1). After additional adjustment for tobacco use and recent illicit drug use, current marijuana use remained significantly and inversely associated with α-Klotho levels (β= -0.084; 95% CI: -0.126 – -0.041; p<0.001). In the combined exposure effect analysis, current marijuana use alone was associated with lower α-Klotho levels compared to non-users (β= -0.087; 95% CI: -0.151 – -0.023; p=0.003), with the greatest reduction observed among those using both marijuana and tobacco (β= -0.112; 95% CI: -0.162 – -0.062; p<0.001), indicating a potential additive effect.

**Table 1:** Weighted regression results for the independent and combined exposure effects of marijuana use with tobacco on α-Klotho levels among adults aged 40–59 years, NHANES 2007–2016 (N=6601)

	*Total n*	*Model 0*		*Model 1*		*Model 2*		*Model 3*	
		*β coefficient (95% CI)*	*p*	*β coefficient (95% CI)*	*p*	*β coefficient (95% CI)*	*p*	*β coefficient (95% CI)*	*p*
**Independent effects of marijuana use**									
Never ®	3271								
Ever	2731	-0.033 (-0.052 – -0.014)	<0.001	-0.031 (-0.050 – -0.012)	0.002	-0.026 (-0.046 – -0.005)	0.017	-0.020 (-0.040–0.001)	0.066
Current	599	-0.100 (-0.144 – -0.057)	<0.001	-0.095 (-0.137 – -0.053)	<0.001	-0.100 (-0.143 – -0.057)	<0.001	-0.084 (-0.126 – -0.041)	<0.001
Combined exposure effects with tobacco use*									
Neither ®	4495								
Marijuana	212	-0.087 (-0.160 – -0.015)	0.019	-0.078 (-0.148 – -0.009)	0.028	-0.088 (-0.152 – -0.024)	0.009	-0.087 (-0.151 – -0.023)	0.009
Tobacco	1507	-0.058 (-0.085 – -0.032)	<0.001	-0.058 (-0.085 – -0.031)	<0.001	-0.057 (-0.088 – -0.026)	<0.001	-0.057 (-0.088 – -0.026)	<0.001
Both	387	-0.102 (-0.142 – -0.061)	<0.001	-0.102 (-0.143 – -0.061)	<0.001	-0.113 (-0.161 – -0.065)	<0.001	-0.112 (-0.162 – -0.062)	<0.001

® Reference categories.

* In the combined exposure effects analysis, marijuana use was dichotomized as current use vs non-use, with both never and ever users categorized as non-users.

Model 0: unadjusted (crude). Model 1: adjusted for survey cycle, age, sex, and race/ethnicity (non-Hispanic White, non-Hispanic Black, Hispanic, and Other). Model 2: adjusted as for Model 1 plus education level (<9, 9–12, and >12 years), Marital status (never married, married/cohabiting, and widowed/divorced/separated), annual family income (<$20000, ≥$20000), current alcohol consumption (yes, no), physical activity (inactive, moderate, vigorous), body mass index (<25, 25–30, and >30 kg/m^2^), estimated glomerular filtration rate (mL/min/1.73 m²), glycohemoglobin (%), uric acid (mg/dL), total cholesterol (mg/dL), systolic blood pressure (mmHg), past 30 days’ prescription medications use (yes, no), depressive symptoms (evaluated by Patient Health Questionnaire-9), and self-reported physician-diagnosed conditions [hypertension, cardiovascular diseases (hypercholesterolemia, congestive heart failure, coronary heart disease, angina, heart attack, or stroke), diabetes, cancer, arthritis, liver diseases, weak/failing kidneys, and respiratory diseases (bronchitis, asthma, or emphysema)]. Model 3: adjusted as for Model 2 plus current tobacco use (yes, no) and current illicit drug use (yes, no). In the combination analysis, adjustment was made only for illicit drug use.

**Figure 1:**
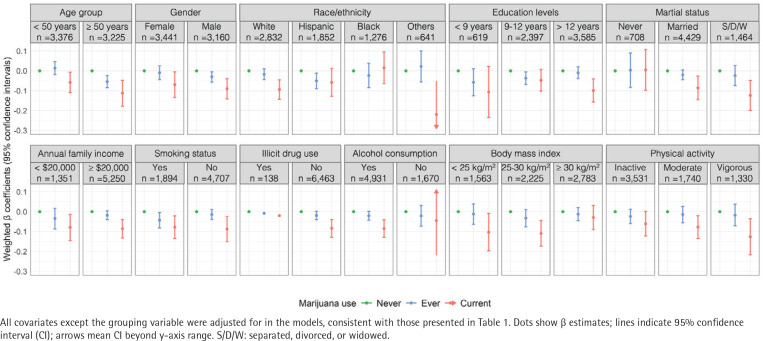
Associations of marijuana use with α-Klotho in subgroup population

Subgroup analyses revealed that the inverse association between marijuana use and α-Klotho levels was more pronounced among non-Hispanic Whites, individuals with a higher level of education, those currently or previously married, current alcohol users, non-obese individuals, and those engaging in regular physical activity (Figure 1). The negative association remained consistent across subgroups defined by age, gender, annual family income, and smoking status. Notably, the association was significant among both current smokers (β= -0.078; 95% CI: -0.134 – -0.021; p=0.008) and non-smokers (β= -0.087; 95% CI: -0.151 – -0.002; p=0.008). Formal tests for multiplicative interactions between marijuana use and each stratification variable showed no statistically significant interaction effects (all p for interaction >0.05; Supplementary file Table 2).

## DISCUSSION

In this nationally representative cohort, we found that current marijuana use was significantly associated with lower circulating α-Klotho levels, even after adjusting for tobacco use, recent illicit drug use, and other potential confounders. Considering that marijuana users are generally more likely to smoke cigarettes and use other psychoactive substances, tobacco use and illicit drug use were included as key covariates in the main analysis. Additionally, we assessed the combined exposure effect of marijuana and tobacco use, and conducted subgroup analyses of marijuana use by smoking status. These analytical results consistently showed that marijuana use was significantly negatively associated with α-Klotho levels, regardless of smoking status. As α-Klotho levels were log-transformed, the β coefficients represent changes on the log scale and indicate the direction and strength of associations. This finding supports our hypothesis and suggests a possible link between cannabis exposure and accelerated biological aging. Our findings are consistent with a recent small-sample study conducted in an obstructive lung disease cohort, which reported that cannabis smoking was associated with accelerated epigenetic aging, particularly as measured by DNAmGrimAge and DNAmPhenoAge^8^. These findings raise concerns about the systemic effects of marijuana, especially in the context of biological aging.

The underlying biological mechanisms linking cannabis use to reduced α-Klotho levels remain speculative but may involve multiple interconnected pathways. Cannabis use may increase exposure to a wide range of environmental toxicants, including heavy metals, polycyclic aromatic hydrocarbons (PAHs), volatile organic compounds (VOCs), and other neuroactive substances, which may directly or indirectly influence circulating levels of α-Klotho. Wei et al.^9^ reported that urinary concentrations of polycyclic aromatic hydrocarbons (PAHs) and volatile organic compounds (VOCs) were significantly higher in cannabis users compared to non-users. Similarly, McGraw et al.^10^ identified elevated levels of heavy metals, particularly cadmium and lead, in both urine and blood samples of cannabis users. Notably, higher internal burdens of these toxicants have each been independently linked to reduced circulating levels of α-Klotho^11-13^. In addition to toxicant exposure, cannabinoids such as Δ9-tetrahydrocannabinol (THC) and cannabidiol (CBD) interact with the endocannabinoid system, which plays a key role in regulating DNA damage and repair, mitochondrial function, and cellular senescence^14,15^. Li et al.^14^ found that CBD disrupts DNA repair pathways and activates p53-mediated cellular senescence in human Sertoli cells^14^. Zhou et al.^15^ showed that activation of CB2 receptors impairs mitochondrial integrity and promotes tubular cell senescence through β-catenin signaling in aged murine kidneys. Although these mechanisms have yet to be fully validated in human studies, they provide a biologically plausible framework through which chronic cannabis use may contribute to decreased α-Klotho levels and the acceleration of systemic aging processes.

### Limitations

Several limitations of this study should be acknowledged. First, due to the cross-sectional nature of NHANES, both marijuana use, tobacco use and serum α-Klotho levels were measured at a single time point, precluding causal inference and limiting the ability to establish temporal relationships. Second, although extensive covariates were adjusted for in our models, the possibility of residual confounding from unmeasured environmental or genetic factors cannot be ruled out. Third, the analytic sample was restricted to adults aged 40–59 years, which limits the generalizability of our findings to younger or older populations. Finally, while NHANES is a nationally representative and ethnically diverse US survey, generalizing these findings to other countries should be done with caution, given international differences in cannabis formulations, routes of administration, and sociolegal contexts. Nonetheless, these limitations highlight the need for longitudinal research to further explore the biological pathways through which cannabis use may influence aging biomarkers like α-Klotho. Given the increasing legalization and use of marijuana, these findings have public health implications for monitoring and mitigating its potential long-term health effects.

## CONCLUSIONS

We found that marijuana use in adults was associated with lower circulating α-Klotho levels independently of tobacco and substance use, suggesting a potential link between cannabis exposure and accelerated biological aging.

## Supplementary Material



## Data Availability

The data supporting this research are available from the following source: https://wwwn.cdc.gov/nchs/nhanes/
