# Supplementary material for: Current marijuana use is associated with lower circulating α-Klotho levels in US adults even after adjusting for tobacco use: A national cross-sectional analysis of NHANES
Source: Tob Induc Dis. 2025 Jul 25;23:10.18332/tid/208001. doi: 10.18332/tid/208001 (PMC12292049; doi:10.18332/tid/208001)
Supplement: Supplementary file 1 [file TID-23-104-s1.pdf]

Supplementary Method

α-Klotho measurement

Frozen blood samples (-80°C) were obtained from participants in NHANES 2007-2016 (five cycles), who provided consent for their samples to be used in future research. During the period 2019–2020, serum klotho was tested by a commercially available and extensively validated ELISA kit produced by IBL International, Japan [1]. The samples were analyzed in duplicate, and the average of the two values was used to calculate the final value. The detailed testing procedure and laboratory quality assurance can be found in the Data Documentation.

Definition of covariates

Sociodemographic characteristics (age, sex, race/ethnicity, marital status, educational level, and family income), lifestyle variables (smoking status, alcohol consumption, and physical activity), disease information were self-reported via computer-assisted questionnaires. Physical examination and blood or urine specimens for laboratory test were performed in the mobile examination center (MEC). Educational level was divided into three categories according to the number of years of education received: less than 9 years, 9 to 12 years, and more than 12 years. Marital status is categorized as: never married, married or cohabiting, divorced/widowed/separated. Family income was divided into two categories based on whether they exceed $20 000 per year. Physical activity was categorized as vigorous, moderate, and inactive [2]. Cardiovascular diseases (CVDs) were defined as the presence of at least one self-report disease, including hypercholesterolemia, congestive heart failure, coronary heart disease, angina, heart attack and stroke. The estimated glomerular filtration rate (eGFR) was calculated using creatinine equation of the Chronic Kidney Disease Epidemiology Collaboration [3]. BMI was calculated as weight in kilograms divided by the square of height in meters, and is categorized as normal (< 25 kg/m^2), overweight (≥ 25 and < 30 kg/m^2), and obesity (≥ 30 kg/m^2) according to American College of Cardiology/American Heart Association guideline [4]. Depressive symptoms were assessed using the 9-item Patient Health Questionnaire (PHQ-9), a widely validated and reliable instrument for depression screening and severity assessment [5].

References:

1. Yamazaki Y, Imura A, Urakawa I, et al. Establishment of sandwich ELISA for soluble alpha-Klotho measurement: Age-dependent change of soluble alpha-Klotho levels in healthy subjects. Biochem Biophys Res Commun. Jul 30 2010;398(3):513-8. doi:10.1016/j.bbrc.2010.06.110
2. Piercy KL, Troiano RP, Ballard RM, et al. The Physical Activity Guidelines for Americans. Jama. Nov 20 2018;320(19):2020-2028. doi:10.1001/jama.2018.14854
3. Lees JS, Welsh CE, Celis-Morales CA, et al. Glomerular filtration rate by differing measures, albuminuria and prediction of cardiovascular disease, mortality and end-stage kidney disease. Nat Med. Nov 2019;25(11):1753-1760. doi:10.1038/s41591-019-0627-8
4. Jensen MD, Ryan DH, Apovian CM, et al. 2013 AHA/ACC/TOS guideline for the management of overweight and obesity in adults: a report of the American College of Cardiology/American Heart Association Task Force on Practice Guidelines and The Obesity Society. Circulation. Jun 24 2014;129(25 Suppl 2):S102-38. doi:10.1161/01.cir.0000437739.71477.ee
5. Kroenke K, Spitzer RL, Williams JB. The PHQ-9: validity of a brief depression severity measure. J Gen Intern Med. Sep 2001;16(9):606-13. doi:10.1046/j.1525-1497.2001.016009606.x

Supplementary Table 1. Characteristics of participants according marijuana use in US adults in NHANES 2005-2016 (n=6,601) *.

	Total	Never	Ever	Current	P for groups
Unweighted sample size	6601	3271	2731	599	
Age (Years, median [IQR])	49 [45, 54]	49 [44, 54]	50 [45, 54]	49 [44, 54]	0.016
Age category (%)					0.166
40-50 years	3743 (55.9)	1858 (57.2)	1507 (54.2)	378 (58.7)	
50-59 years	2858 (44.1)	1413 (42.8)	1224 (45.8)	221 (41.3)	
Sex (%)					<0.001
Men	3160 (48.4)	1367 (41.7)	1444 (51.8)	349 (59.1)	
Women	3441 (51.6)	1904 (58.3)	1287 (48.2)	250 (40.9)	
Race/ethnicity (%)					<0.001
non-Hispanic White	2832 (71.6)	941 (58.6)	1558 (81.2)	333 (76.5)	
Hispanics	1852 (12.8)	1343 (22.4)	426 (6.3)	83 (6.6)	
non-Hispanic Black	1276 (9.6)	540 (10.0)	579 (8.6)	157 (12.8)	
Other	641 (6.0)	447 (9.0)	168 (3.9)	26 (4.1)	
Education levels (%)					<0.001
< 9 years	619 (4.6)	499 (8.4)	92 (1.9)	28 (2.8)	
9-12 years	2397 (31.7)	1177 (33.1)	935 (28.9)	285 (40.9)	
>12 years	3585 (63.6)	1595 (58.5)	1704 (69.2)	286 (56.3)	
Marital status (%)					<0.001
Never married	708 (9.1)	300 (8.3)	311 (8.9)	97 (13.4)	
Married	4429 (71.3)	2357 (75.7)	1750 (69.5)	322 (61.5)	
S/D/W	1464 (19.6)	614 (15.9)	670 (21.5)	180 (25.1)	
Annual family income (%)					<0.001
≤ $20,000 per year	1351 (12.6)	645 (13.6)	493 (10.0)	213 (22.3)	
> $20,000 per year	5250 (87.4)	2626 (86.4)	2238 (90.0)	386 (77.7)	
Tobacco use (%)					<0.001
No	4707 (72.8)	2711 (84.4)	1784 (69.3)	212 (41.5)	
Yes	1894 (27.2)	560 (15.6)	947 (30.7)	387 (58.5)	
Illicit drug use (%)					<0.001
No	6463 (98.5)	3253 (99.6)	2680 (99.0)	530 (91.4)	
Yes	138 (1.5)	18 (0.4)	51 (1.0)	69 (8.6)	
Alcohol consumption (%)					<0.001
No	1670 (19.3)	1307 (35.7)	317 (8.9)	46 (4.3)	

Yes	4931 (80.7)	1964 (64.3)	2414 (91.1)	553 (95.7)	
Physical activity (%)					0.044
Inactive	3531 (47.4)	1839 (49.9)	1359 (45.5)	333 (47.1)	
Moderate	1740 (29.3)	844 (29.0)	729 (28.9)	167 (32.6)	
Vigorous	1330 (23.3)	588 (21.1)	643 (25.6)	99 (20.2)	
Body mass index (%)					<0.001
< 25 kg/m2	1563 (24.9)	744 (23.7)	627 (24.3)	192 (32.7)	
25-30 kg/m2	2255 (35.1)	1115 (32.9)	937 (36.9)	203 (35.5)	
≥ 30 kg/m2	2783 (40.0)	1412 (43.5)	1167 (38.8)	204 (31.8)	
Hypertension (%)					0.335
No	4282 (67.1)	2172 (65.9)	1731 (67.6)	379 (69.1)	
Yes	2319 (32.9)	1099 (34.1)	1000 (32.4)	220 (30.9)	
Cardiovascular diseases (%)					0.169
No	6145 (94.4)	3087 (95.1)	2522 (94.0)	536 (93.2)	
Yes	456 (5.6)	184 (4.9)	209 (6.0)	63 (6.8)	
Diabetes (%)					<0.001
No	5798 (90.6)	2819 (88.4)	2436 (91.9)	543 (93.4)	
Yes	803 (9.4)	452 (11.6)	295 (8.1)	56 (6.6)	
Arthritis (%)					<0.001
No	4890 (73.2)	2565 (76.5)	1925 (71.8)	400 (66.4)	
Yes	1711 (26.8)	706 (23.5)	806 (28.2)	199 (33.6)	
Respiratory diseases (%)					<0.001
No	5451 (82.8)	2810 (85.7)	2190 (81.7)	451 (76.2)	
Yes	1150 (17.2)	461 (14.3)	541 (18.3)	148 (23.8)	
Liver diseases (%)					0.009
No	6254 (95.3)	3121 (96.4)	2572 (94.9)	561 (92.5)	
Yes	347 (4.7)	150 (3.6)	159 (5.1)	38 (7.5)	
Weak/failing kidneys (%)					0.955
No	6447 (98.1)	3195 (98.2)	2670 (98.1)	582 (98.1)	
Yes	154 (1.9)	76 (1.8)	61 (1.9)	17 (1.9)	
Cancer (%)					0.001
No	6193 (91.8)	3113 (93.7)	2523 (90.4)	557 (90.9)	
Yes	408 (8.2)	158 (6.3)	208 (9.6)	42 (9.1)	
Prescription medications use (%)					0.018
No	3772 (61.7)	1740 (59.7)	1690 (63.8)	342 (59.1)	
Yes	2827 (38.3)	1530 (40.3)	1040 (36.2)	257 (40.9)	

Depressive symptoms	2 [0, 4]	1 [0, 4]	2 [0, 5]	3 [1, 7]	<0.001
eGFR (mL/min/1.73 m²)	94.88 (15.96)	96.25 (16.65)	93.39 (15.34)	94.17 (15.89)	0.001
Glycohemoglobin (%)	5.85 (1.01)	5.93 (1.09)	5.79 (0.95)	5.74 (0.90)	<0.001
Uric acid (mg/dL)	5.4 (1.38)	5.29 (1.37)	5.51 (1.37)	5.52 (1.42)	0.004
Total cholesterol (mg/dL)	203.18 (42.22)	202.92 (40.57)	203.22 (41.46)	204.44 (51.64)	0.038
SBP (mmHg)	123.15 (15.74)	123.22 (16.09)	122.8 (15.43)	124.32 (15.81)	0.058

* Weighted means (standard deviations, SDs), weighted medians (interquartile ranges, IQRs), and numbers (weighted percentages) were used to present the baseline characteristics of the study participants where appropriate. Differences between the groups were tested using ANOVA, the Kruskal–Wallis test, or Fisher's exact test when appropriate.

Abbreviations: eGFR, estimated Glomerular Filtration Rate; SBP, Systolic blood pressure

Supplementary Table 2. Stratified associations between marijuana use and serum α-Klotho levels, with corresponding p-values for interaction.

Subgroup	Marijuana Use	Effect (Beta [95% CI])	Interaction P
**Age group**			**0.55**
<50Years	Ever	0.0135 (-0.0187, 0.0458)	
	Current	-0.0581 (-0.1091, -0.0071)	
≥50Years	Ever	-0.0539 (-0.0846, -0.0233)	
	Current	-0.1121 (-0.1771, -0.0471)	
**Sex**			**0.66**
Female	Ever	-0.0094 (-0.0443, 0.0255)	
	Current	-0.0692 (-0.1338, -0.0047)	
Male	Ever	-0.0303 (-0.0562, -0.0043)	
	Current	-0.0898 (-0.1402, -0.0394)	
**Race/ethnicity**			**0.17**
non-Hispanic White	Ever	-0.0172 (-0.0442, 0.0098)	
	Current	-0.0939 (-0.1428, -0.0451)	
Hispanics	Ever	-0.0500 (-0.0889, -0.0111)	
	Current	-0.0578 (-0.1279, 0.0124)	
non-Hispanic Black	Ever	-0.0230 (-0.0843, 0.0383)	
	Current	0.0151 (-0.0646, 0.0949)	
Other	Ever	0.0221 (-0.0556, 0.0998)	
	Current	-0.2194 (-0.3890, -0.0498)	
**Education level**			**0.51**
< 9 years	Ever	-0.0573 (-0.1249, 0.0102)	
	Current	-0.1065 (-0.2344, 0.0215)	
9–12 years	Ever	-0.0370 (-0.0691, -0.0049)	
	Current	-0.0471 (-0.1015, 0.0072)	
> 12 years	Ever	-0.0099 (-0.0392, 0.0194)	
	Current	-0.0986 (-0.1570, -0.0402)	
**Marital status**			**0.54**
Never married	Ever	0.0037 (-0.0826, 0.0900)	
	Current	0.0048 (-0.0973, 0.1069)	
Married or cohabiting	Ever	-0.0203 (-0.0445, 0.0040)	
	Current	-0.0858 (-0.1446, -0.0269)	
Widowed, divorced, or separated	Ever	-0.0237 (-0.0746, 0.0271)	
	Current	-0.1234 (-0.1993, -0.0475)	
**Annual family income**			**0.66**
≤ $20,000/year	Ever	-0.0344 (-0.0863, 0.0176)	
	Current	-0.0787 (-0.1444, -0.0130)	
> $20,000/year	Ever	-0.0177 (-0.0399, 0.0044)	
	Current	-0.0855 (-0.1318, -0.0392)	

**Tobacco use**			**0.18**
Yes	Ever	-0.0430 (-0.0816, -0.0044)	
	Current	-0.0777 (-0.1344, -0.0210)	
No	Ever	-0.0140 (-0.0384, 0.0104)	
	Current	-0.0873 (-0.1507, -0.0238)	
**Illicit drug use**			**0.86**
Yes	Ever	-0.0079 (NA, NA)	
	Current	-0.0202 (NA, NA)	
No	Ever	-0.0190 (-0.0400, 0.0020)	
	Current	-0.0836 (-0.1280, -0.0392)	
**Alcohol consumption**			**0.52**
Yes	Ever	-0.0203 (-0.0426, 0.0019)	
	Current	-0.0849 (-0.1285, -0.0412)	
No	Ever	-0.0210 (-0.0731, 0.0311)	
	Current	-0.0450 (-0.2190, 0.1290)	
**Body mass index**			**0.11**
≤ 25 kg/m^2	Ever	-0.0120 (-0.0635, 0.0395)	
	Current	-0.1026 (-0.1972, -0.0079)	
25~30 kg/m^2	Ever	-0.0328 (-0.0758, 0.0102)	
	Current	-0.1090 (-0.1733, -0.0439)	
≥ 30 kg/m^2	Ever	-0.0123 (-0.0451, 0.0205)	
	Current	-0.0294 (-0.0899, 0.0310)	
**Physical activity**			**0.093**
Inactive	Ever	-0.0240 (-0.0591, 0.0110)	
	Current	-0.0604 (-0.1216, 0.0009)	
Moderate	Ever	-0.0140 (-0.0551, 0.0271)	
	Current	-0.0772 (-0.1350, -0.0195)	
Vigorous	Ever	-0.0171 (-0.0715, 0.0373)	
	Current	-0.1260 (-0.2160, -0.0359)	

Supplementary Table 3. STROBE checklist.

Item No.	Recommendation	Location in Manuscript
**Title and Abstract**		
1	Indicate the study’s design with a commonly used term in the title or abstract	Title, Abstract
2	Provide in the abstract an informative and balanced summary of what was done and what was found	Abstract
**Introduction**		
3	Explain the scientific background and rationale for the investigation being reported	Introduction, Paragraphs 1
4	State specific objectives, including any prespecified hypotheses	Introduction, Paragraphs 2
**Methods**		
5	Describe the setting, locations, and relevant dates, including periods of recruitment, exposure, follow-up, and data collection	Methods > Data source and participants
6	Give the eligibility criteria, and the sources and methods of selection of participants	Methods > Data source and participants
7	Clearly define all outcomes, exposures, predictors, potential confounders, and effect modifiers. Give diagnostic criteria, if applicable	Methods > Measures, Supplementary methods
8	For each variable of interest, give sources of data and details of methods of assessment (measurement). Describe comparability of assessment methods if there is more than one group	Methods > Measures, Supplementary methods
9	Describe any efforts to address potential sources of bias	Methods > Statistical Analysis; Discussion, paragraph 2
10	Explain how the study size was arrived at	Methods > Data source and participants
11	Explain how quantitative variables were handled in the analyses. If applicable, describe which groupings were chosen and why	Methods > Measures and Statistical Analysis
12	Describe all statistical methods, including those used to control for confounding	Methods > Statistical Analysis

Item No.	Recommendation	Location in Manuscript
13	Describe any methods used to examine subgroups and interactions	Methods > Statistical Analysis
14	Explain how missing data were addressed	Methods > Statistical Analysis
15	If applicable, describe analytical methods taking account of sampling strategy	Methods > Statistical Analysis
16	Describe sensitivity analyses	Not applicable (N/A)
**Results**		
17	Report numbers of individuals at each stage of study—e.g., numbers potentially eligible, examined for eligibility, confirmed eligible, included in the study, completing follow-up, and analysed	Methods > Data source and participants; Results, first paragraph
18	Give characteristics of study participants (e.g., demographic, clinical, social) and information on exposures and potential confounders	Results, first paragraph; Supplementary Table 1
19	Indicate number of participants with missing data for each variable of interest	Addressed via multiple imputation; Methods > Statistical Analysis
20	Report numbers of outcome events or summary measures	Results > Table
21	Give unadjusted estimates and, if applicable, confounder-adjusted estimates and their precision (e.g., 95% confidence interval). Make clear which confounders were adjusted for and why they were included	Results > Table and text
22	Report category boundaries when continuous variables were categorized	Methods > Measures and Statistical Analysis; Table footnotes
23	If relevant, consider translating estimates of relative risk into absolute risk for a meaningful time period	Not applicable (N/A)
**Discussion**		
24	Summarise key results with reference to study objectives	Discussion, paragraph 1
25	Discuss limitations of the study, taking into account sources of potential bias or imprecision. Discuss both direction and magnitude of any potential bias	Discussion, paragraph 4 and 5

Item No.	Recommendation	Location in Manuscript
26	Give a cautious overall interpretation of results considering objectives, limitations, multiplicity of analyses, results from similar studies, and other relevant evidence	Discussion, paragraph 3 and final paragraph
27	Discuss the generalisability (external validity) of the study results	Discussion, last paragraph
**Other information**		
28	Give the source of funding and the role of the funders for the present study and, if applicable, for the original study on which the present article is based	Funding section
29	Give the name of the ethical committee that approved the study and the study's reference number	Methods > Data source and participants

Note: This checklist was adapted from the STROBE statement: von Elm E, Altman DG, Egger M, et al. The Strengthening the Reporting of Observational Studies in Epidemiology (STROBE) Statement: Guidelines for reporting observational studies. *Lancet*. 2007;370(9596):1453–1457.

© 2025 Wei K. and Chen X.
